# Epidemic Jaundice in the Mediterranean Expeditionary Force

**Published:** 1916-07-15

**Authors:** 


### EPIDEMIC JAUNDICE IN THE MEDITERRANEAN EXPEDITIONARY FORCE.

### Research under Difficulties.

The ill-fated Dardanelles campaign is so much a matter of ancient history that many people have resolutely put it out of their minds altogether, because of its unpleasant associations of failure: failure predestined and inevitable from the first, in spite of the magnificent courage and heroism of the subordinate commanders and of the rank and file, British and Colonial. The medical lessons of this undertaking, however, are less provocative of melancholy reflections than the military; for though epidemic disease was undoubtedly very widespread on the Peninsula, there can be no doubt that the great care paid to sanitation in the extremely difficult local conditions was responsible for the low mortality rate and for the fact that it was possible to hold the trenches at all.

### Disease at the Dardanelles.

When the medical history of the war comes to be written by those to whom the task has been entrusted, the chapters dealing with the Dardanelles may not improbably prove to be, from a medical as opposed to a surgical standpoint, the most interesting of any. Hitherto a wise reticence has been preserved officially on the subject: wise because a part of our Army is still at Salonika, where very likely similar problems present, or may present, themselves to the enemy as well as to ourselves, and there is no need to let him into our confidence concerning the lessons we have learnt at Gallipoli. Occasionally the results of individual research there performed are published in the medical or scientific journals; and sometimes a discreet hand lifts the curtain on such scenes as may safely be shown without disclosing anything that can assist the enemy. In the latter category may be placed a lecture delivered a short time ago before the Royal Society of Medicine by Dr. L. S. Dudgeon, of St. Thomas's Hospital, on his experiences at Gallipoli and elsewhere as one of the Advisory Committee on Epidemic Diseases and Sanitation. The narrative is personal, in the sense that it contains the impressions and opinions of the observer, not the official conclusion or recommendations of the Committee of which he was a member. It shows very clearly the disadvantages under which much of the medical work was done; for we read of a laboratory where for days at a time no work involving the manipulation of culture media could be attempted because of perpetual dust-storms, and of another so small that two men could only just squeeze into it at the same time. The photograph of the sand-bagged cavern at Cape Helles, which is reproduced in Dr. Dudgeon's paper in the Transactions of the Royal Society of Medicine, shows surely the most extraordinary laboratory that any bacteriologist ever tried to work in.

### Camp Jaundice.

Amongst the epidemic diseases encountered "camp jaundice" is perhaps the most fully considered, as it is one of the most mysterious. This disease began in Egypt in July 1915, and made its first appearance in the Peninsula amongst some French engineers. Curiously, the outbreak was in the beginning suspected of being due to malingering; it was thought that picric acid was being swallowed. A fortnight after the disease had been noticed amongst the French troops, the adjacent British troops began to suffer, and the epidemic soon became general, reaching a maximum in October. It appeared also at Imbros and Lemnos, attacking there men who had never visited the Peninsula. It is believed that the Turks did not catch the infection, which is certainly strange if true, since in some cases the trenches were not ten yards apart. Possibly after the end of the war it may turn out that this alleged immunity of the Turkish troops is fictitious: certainly they suffered severely from many forms of sickness. Fortunately, this camp jaundice was a mild disease, far different from the epidemic jaundice which has infested Alexandria at intervals in the last fifty years, and is frequently fatal. The French bacteriologists were able to isolate from the blood in 112 cases out of 606 an interesting microbe which they hold to be the cause of the trouble. They have christened it provisionally *B. paradardanellensis*, and are still investigating its relationships. Dr. Dudgeon has had some cultures of it from the French, but offers no opinion as to whether they are right or wrong in their conclusions.

### 1900-1915: A Contrast.

Those who had anything to do with the Medical Service in the South African campaign will be struck with the contrast between the treatment of epidemic diseases then and now, only fifteen years later. One-tenth part of the care and trouble now devoted to Army hygiene and sanitation would have saved thousands of lives in the South African Field Force. Epidemic or "camp" jaundice was common enough in the British and Colonial Forces, but no one attempted to work out its etiology or to isolate its organism, so far as we know. Indeed, no organisation existed for collecting the materials, let alone for doing the necessary bacteriological work on them. Yet on that shell-swept beach, at the end of the narrow tongue won by the immortal valour of British and French soldiers, the secret of "camp" jaundice was wrested from Nature, if the researches of Sarrailhé and Clunet are confirmed, in stifling dug-outs and sand-bagged "laboratories" underground. Here is the romance of war indeed.

